# Immunoglobulins and Efgartigimod reduce neuronal activity in primary rat cortical neuron cultures

**DOI:** 10.3389/fncel.2026.1939772

**Published:** 2026-10-28

**Authors:** Felix Marti Bautista, Nilufar Nazarzadeh, Niklas Genseke, Elena Groß, Daniel Navin Olschewski, Gereon Rudolph Fink, Michael Schroeter, Maria Adele Rueger, Sabine Ulrike Vay

**Affiliations:** 1 Department of Neurology, University of Cologne, Faculty of Medicine and University Hospital, Cologne, Germany; 2 Cognitive Neuroscience, Institute of Neuroscience and Medicine (INM-3), Research Centre Juelich, Juelich, Germany

**Keywords:** cortical neuronal network, cortical neurons, Efgartigimod alfa, fc gamma receptor IIb (FcγRIIb), IgG-based therapeutics, immunoglobulin therapy, MEA, microelectrode array

## Abstract

Immunoglobulin-based therapies are increasingly used to modulate neuroinflammatory diseases, yet it remains unclear whether they directly affect neuronal function. Here, we provide evidence that primary rat cortical neurons express both the inhibitory Fc gamma receptor IIb (FcγRIIb) and the neonatal Fc receptor (FcRn), as assessed by immunocytochemistry and mRNA analysis. FcγRIIb expression was upregulated under proinflammatory conditions, indicating inflammation-dependent regulation. Using microelectrode array recordings, we observed that immunoglobulin treatment reduced neuronal activity under both physiological and inflammatory conditions. Similarly, Efgartigimod, a high-affinity FcRn blocker, was associated with a sustained reduction in neuronal activity, with the observed effects suggesting a trend toward dose dependence under control conditions. Together, these findings provide proof-of-concept evidence that primary cortical neuronal cultures respond to immunoglobulins and Efgartigimod *in vitro* and highlight neuronal Fc receptor signaling as a promising avenue for future studies.

## Introduction

Immunoglobulins are key components of the adaptive immune system, predominantly found circulating in the bloodstream (Sun et al., 2020). Their primary functions include neutralizing antigens, promoting phagocytosis via opsonization, activating macrophages, and initiating the complement cascade, all of which contribute to an effective immune response against invading pathogens (Cobb, 2020). Intravenous immunoglobulin (IVIG) therapy has become a well-established treatment modality for a broad spectrum of autoimmune and inflammatory disorders. It is particularly prominent in neurology as a first-line therapy for several immune-mediated neuropathies such as Guillain-Barré syndrome and chronic inflammatory demyelinating polyneuropathy (CIDP), or exacerbations of myasthenia gravis (MG) (Lünemann et al., 2015). The therapeutic efficacy of IVIG is attributed to its broad anti-inflammatory and immunomodulatory effects, which include modulation of Fc receptor function and neutralization of pathogenic autoantibodies, thereby helping to restore immune homeostasis (Lünemann et al., 2015). IVIG preparations primarily consist of immunoglobulin G (IgG) monomers (Galeotti et al., 2017). A key target for IgG via its fragment crystallizable (Fc) region are Fc gamma receptors (FcγRs) of immune cells. The binding of IgG to FcγRs can either activate or inhibit immune responses, depending on the receptor subtype. Activating receptors include FcγRI, FcγRIIa, FcγRIIc, and FcγRIIIa, while FcγRIIb is the only known inhibitory receptor (Bournazos et al., 2020). Conceivably, the FcγRIIb is particularly relevant for mediating the anti-inflammatory effects of IVIG. Although it has low affinity for monomeric IgG, IVIG has been shown to upregulate FcγRIIb expression on effector cells such as B cells, thereby increasing the activation threshold for inflammatory signaling pathways (Lünemann et al., 2015; Anthony et al., 2011; Bruhns et al., 2003; Kaneko et al., 2006; Samuelsson et al., 2001).

Recent studies have demonstrated that specific Fc receptors, including the FcγRIIb, are not only expressed on immunocompetent cells but also on neurons within the central nervous system (CNS) (Fuller et al., 2014; Kam et al., 2013; Stamou et al., 2018). However, the functional relevance of neuronal FcγRIIb remains poorly understood. The expression of these receptors on neurons raises the possibility of a novel mechanism of IVIG action within the CNS, namely through direct interactions between immunoglobulins and neuronal cells. This concept gains further significance in light of evidence showing that IVIG cross the blood–brain barrier and reach the CNS (Guenther and Neu, 1984; Magga et al., 2010; Puli et al., 2014; St-Amour et al., 2013). Building on this observation, it is important to investigate whether immunoglobulins directly interact with neurons and to further elucidate the potential role of neuronal Fc receptors. In this regard, Fc receptor-targeting therapies such as Efgartigimod have emerged as an alternative strategy to selectively reduce pathogenic IgG levels. Efgartigimod alfa, an IgG1-derived Fc fragment, is an approved drug for patients with MG and has demonstrated beneficial effects in CIDP as well, by competing with endogenous IgG for binding to the neonatal Fc receptor (FcRn) (Ulrichts et al., 2018; Vaccaro et al., 2005). Although FcRn was first identified for its role in maternal IgG transport across the placenta, it is now recognized to play a critical function in adults by recycling IgG in endothelial and immune cells, thereby extending its half-life and modulating immune responses (Roopenian and Akilesh, 2007; Baker et al., 2014). Disruption of this recycling pathway by Efgartigimod reduces circulating autoantibody levels, thereby mitigating disease activity in antibody-mediated autoimmune conditions such as MG and CIDP (Roopenian and Akilesh, 2007; Lünemann, 2021, 2025). Given the emerging evidence that Fc receptors may play a role beyond classical immune functions—particularly within the CNS—it is crucial to investigate their expression and function in neuronal populations.

The primary aim of this study was to investigate the expression of neuronal Fc receptors and their association with neuronal network responses to immunoglobulins and Efgartigimod —an idea that challenges the classical paradigm of antibody–receptor interactions being limited to immune cells. We hypothesized that FcγRIIb and FcRn expression in primary cortical neurons is influenced by the inflammatory milieu and that neuronal activity is altered following exposure to immunoglobulins and Efgartigimod under both physiological and inflammatory conditions. To test these hypotheses, we employed a combination of immunocytochemistry, RT-qPCR, and microelectrode array (MEA) technology to analyze both receptor expression and neuronal network responses under different experimental conditions in primary cell culture.

## Materials and methods

### Glia cell culture

All animal procedures were carried out in accordance with the German Laws for Animal Protection and approved by the local animal care committee (Tierschutz-Beauftragte University of Cologne) and local governmental authorities (AZ UniKöln_Anzeige §4.21.015). Animal procedures for tissue harvesting of primary cortical neurons were obtained from the Institute of Biological Information Processing (IBI-2), Department of Mechanobiology, Research Center Juelich (Animal testing license: 84-02.04.2015.A173, LANUV NRW, Germany).

Wistar rats for breeding were maintained in polysulfone cages with sawdust bedding on a 12 h light/dark cycle. Food and water were provided ad libitum in pathogen-free conditions.

Pure neonatal microglia cultures were harvested from the cortices of neonatal Wistar rats (P0–P3), which were sacrificed by unanesthetized decapitation (Vay et al., 2021). Briefly, cortices were dissected, and blood vessels, meninges, and white matter removed. Consecutively, the cortical tissue was cut into small pieces and incubated in 0.05% trypsin/0.02% EDTA solution (Thermo Fisher Scientific, Waltham, USA) for 15 min at 37 °C. The addition of cell culture media (Dulbecco's essential media (DMEM) containing 10% fetal calf serum (FCS), 1% penicillin/streptomycin, and 2 mM l-glutamine) (Thermo Fisher Scientific, Waltham, USA) neutralized the reaction. The cortices were dissociated by up- and down-pipetting. The resulting cell suspension was centrifuged at 1,200 rpm for 2 min, and the cell pellets were resuspended in culture media and grown at 37 °C with 5% CO_2_. The culture medium was changed on the third day.

### Microglia harvest

After 8-10 days, cultures were shaken for 1 h at 250 rpm on an orbital shaker (37 °C) to detach microglia. The medium containing the layer of detached microglia was collected and immediately centrifuged at 1,200 rpm for 2 min. After removing the supernatant, the obtained microglia pellet was resuspended in fresh culture medium and seeded into subcultures for further experiments (see below). Flasks were refilled with culture medium, and microglia harvesting was repeated three times at intervals of 3 days.

### Microglia treatment

Twenty-four h after seeding in subcultures, microglia were exposed to 10 ng/ml Lipopolysaccharides (LPS) to induce an inflammatory phenotype (Vay et al., 2018). LPS-treated microglia secrete various proinflammatory mediators, including cytokines, chemokines, and reactive oxygen species, thereby modulating their microenvironment (Orihuela et al., 2016). In other microglia, a simple medium change was performed. Twenty-four h after stimulation, the conditioned medium from the LPS-treated and the untreated microglia was obtained, and both were used in further experiments (see below).

### Primary cortical neurons

Animal procedures for tissue harvesting of primary cortical neurons were obtained from the Institute of Biological Information Processing (IBI-2), Department of Mechanobiology, Research Center Juelich (Animal testing license: 84-02.04.2015.A173, LANUV NRW, Germany). Embryonic rat cortical neurons were obtained from pregnant rats at 18-19 days of gestation (Abraham et al., 2019). Cortices were isolated from embryonic brains and further trypsinized using 0.05% trypsin/0.02% EDTA solution (Thermo Fisher Scientific, Waltham, USA) for 15 min at 37 °C. The tissue was transferred to prewarmed neurobasal media (Thermo Fisher Scientific, Waltham, USA), supplemented with 1% GlutaMAX (Thermo Fisher Scientific, Waltham, USA), 2% B-27 (Thermo Fisher Scientific, Waltham, USA), and 0.1% Gentamicin (Sigma, Taufkirchen, Germany). Cortices were washed three times to remove any remaining trypsin solution. Consequently, cortical cells were dissociated by trituration. After cell counting 200,000 neurons were seeded in 1 ml and 500 μl of neurobasal media, respectively on microelectrode arrays (60MEA200/30iR-T, Multi Channel Systems (MCS), Reutlingen, Germany) and 24-well-plates, which were both pre-coated with 15% poly-l-ornithine diluted with ultrapure water (Sigma, Taufkirchen, Germany) and 1.25% fibronectin diluted with 1 × PBS (R&D Systems, Minneapolis, USA). Half of the medium was changed every 2 days.

### Microelectrode arrays (MEAs)

Cortical neurons were cultured for 15-20 days until mature bursting and synchronic activity were achieved in at least 10 electrodes within the network. For the experiments, the neurons were treated with 5 mg/ml immunoglobulins (Gamunex, Grifols, Barcelona, Spain), 1.8 mg/ml or 3.6 mg/ml Efgartigimod alfa (argenx BV, Ghent, Belgium) or 250 μl of conditioned medium derived from LPS-treated microglia or untreated microglia. A naive control group received a treatment with 250 μl of neuronal or microglial medium. Measurements were performed shortly before stimulation (baseline), immediately (acute) and at 1 h, 4 h, and 24 h after stimulation. In the co-stimulation experiments, neurons were first exposed to conditioned medium from LPS-treated microglia to establish a proinflammatory environment. One h later, the cells were further stimulated with immunoglobulins.

To record cortical network activity, we used a commercially available 60MEA200/30iR-T (Multi Channel Systems (MCS), Reutlingen, Germany) and a 60-channel MEA filter-amplifier (MEA2100-Mini-HS60, MCS, Reutlingen, Germany) both connected to an interface board (MCS-IFB 3.0 Multiboot Interface Board, MCS, Reutlingen, Germany). All recordings were conducted in an incubator at 37 °C and 5% CO_2_. Raw signals were recorded using a software user interface (MC Experimenter, MCS, Reutlingen, Germany) and analyzed offline (MC Analyzer, MCS, Reutlingen, Germany). All signals were filtered by a second-order Bessel high-pass filter (cut-off at 200 Hz). Spikes represent the spontaneous activity of cortical neurons at respective electrodes (Tanskanen et al., 2018). The spikes in the filtered data streams were detected using a negative threshold (-4.5 StDev of the peak-to-peak noise) (Habibey et al., 2017; Hyvärinen et al., 2019). The number of spikes within a 5-min time interval was analyzed. The mean number of spikes per second and timestamps of spikes recorded from individual electrodes were extracted by the MC_Analyzer and analyzed statistically (GraphPad Prism Version 11.1.0, GraphPad Software Inc., San Diego, CA, USA). Finally, changes of neuronal activity were analyzed by normalizing the spike rate at each measurement time point to the respective baseline spike rate. The acute, 1 h, 4 h, and 24 h time points were predefined as part of the experimental design and analyzed separately. MEA experiments were performed using neuronal cultures derived from at least three independent neuronal preparations. Neuronal activity was recorded from multiple active electrodes per well. Electrode-level measurements were averaged within each well to obtain a single well-level value, which was used for statistical analysis. Mean values ± SEM were established among equally treated wells.

### Treatment of neurons for RT-qPCR and Immunocytochemical staining

For further experiments, neurons seeded in 24-well-plates were exposed to an inflammatory milieu by treating them with 205 μl of the conditioned medium from LPS-treated microglia for 24 h. Neurons of the control group were treated with unconditioned microglia medium.

### Real-time quantitative PCR (RT-qPCR)

RNA from cultivated cortical neurons was isolated using tissue and cell lysis solution and proteinase K (Lucigen Corporation, Biosearch Technologies, Middleton, WI, USA). RNA concentration and purity were evaluated photometrically (FLUOstar Omega, BMG LAB-TECH, Ortenberg, Germany). According to the manufacturer's recommendations, the total RNA (10 ng) was converted to cDNA by reverse transcription with the QuantiTect Transcription Kit (Qiagen, Hilden, Germany). All primers used in this study were obtained from Biolegio (Nijmegen, The Netherlands). Primer sequences and PCR parameters are listed in Table 1. The samples were amplified and quantified on a LightCycler 96 (Roche, Mannheim, Germany). PCR product integrity was evaluated by melting point analysis and agarose gel electrophoresis. The threshold cycle (CT) was normalized to housekeeping ribosomal protein L13a (RPL13a; ΔCT). Data are depicted as 2^(−Δ*ΔCT*)^. RT-qPCR was performed in technical triplets, with at least three independent biological replicates per experimental condition. Mean values ± SEM were calculated for all samples.

**Table 1:** Primers and RT-qPCR parameters.

Primer	Sequences forward/reverse 5‘-3‘	Temperature (°C) step 1/2/3	Duration (s) step 1/2/3	Accession number
Fcgrt (FcRn)	CGG AGC TCA AGT TTC GAT TC/ GGT GTT CAT CTC CAC GTT TG	95/57/72	15/15/45	NM_033351.2
Fcgr2b (FcγRIIb)	TGA GTC TCA TGC AGG TCT TC/ TCC CAT TGT GAA ACC ACT GA	95/57/72	15/15/45	NM_175756.3
RPL13a	TCT CCC AAA GCG GAT GAA CA/ CAA CAC CTT GAG GCG TTC CA	95/57/72	15/15/45	NM_173340.2

The table lists the primers used in this study, including forward and reverse sequences, as well as the annealing temperatures and durations of each cycling step.

### Immunocytochemical staining of neurons

Neuronal cultures were fixed with 4% paraformaldehyde (PFA). All antibodies used in this study are listed in Table 2. For visualization of primary antibodies, fluorescein-labeled anti-mouse immunoglobulin G (IgG) or anti-rabbit IgG were used. All cells were counterstained with DAPI. Representative images were taken using a digital microscope (Keyence BZ-9000E, Osaka, Japan).

**Table 2:** Primary and secondary antibodies.

Antibody	Type	Dilution	Origin
Mouse anti-FcγRIIb	Primary, monoclonal	1:250	BD Biosciences, Cat# 550271
Rabbit anti-FcRn	Primary, polyclonal	1:200	Invitrogen, Cat# PA5-97111
Mouse anti-MAP2	Primary, monoclonal	1:1000	Abcam, Cat# ab11267
Rabbit anti-MAP2	Primary, monoclonal	1:2000	Abcam, Cat# ab254264
Donkey anti mouse IgG (H+L) Alexa Fluor 488	Secondary, polyclonal	1:200	Invitrogen, Cat# A-21202
Donkey anti mouse IgG (H+L) Alexa Fluor 568	Secondary, polyclonal	1:200	Invitrogen, Cat# A10037
Donkey anti rabbit IgG (H+L) Alexa Fluor 488	Secondary, polyclonal	1:200	Invitrogen, Cat# A-21206
Donkey anti rabbit IgG (H+L) Alexa Fluor 568	Secondary, polyclonal	1:200	Invitrogen, Cat# A10042

The table provides an overview of the primary and secondary antibodies employed in this study, including information on dilutions, manufacturers, and catalog numbers.

### Quantitative analysis of Immunocytochemical stainings

For a quantitative analysis of immunocytochemical stainings, somata with specific expression of the receptors FcγRIIb and FcRn were counted manually in at least 30 neurons for each condition, derived from at least three independent experiments. To visualize changes after treatment with the conditioned medium of LPS-treated microglia, the absolute numbers were then normalized to the control group. Mean values ± SEM were calculated for all samples.

### Data processing and statistical analysis

Raw numerical data were processed using Microsoft Excel (Version 16.66.1, Microsoft Corp., Seattle, WA, USA). Images were processed using ImageJ (v.2.0.0 Fiji) by adjusting brightness, contrast, and sharpness for each color channel, with only minimal modifications. Figures were created using Microsoft PowerPoint (Version 16.66.1, Microsoft Corp., Seattle, WA, USA) and BioRender (Science Suite Inc., Toronto, ON, Canada). Statistical analyses and graphical visualization were performed using GraphPad Prism (Version 11.1.0, GraphPad Software Inc., San Diego, CA, USA).

Data distribution was assessed using the Shapiro–Wilk test. As the data were predominantly non-normally distributed, non-parametric statistical tests were used throughout. Comparisons between two experimental groups were performed using two-tailed Mann–Whitney U tests. For comparisons involving three or more groups, Kruskal–Wallis tests followed by Dunn's multiple-comparisons tests were used, with adjusted p values reported for the corresponding pairwise comparisons.

For MEA analyses, electrode-level measurements were averaged within each well to obtain a single well-level value, and statistical analyses were performed using these well-level data. The number of wells (n) included in each experimental condition is indicated in the respective figures. Neuronal cultures used for MEA experiments were derived from at least three independent neuronal preparations. The predefined acute, 1 h, 4 h, and 24 h MEA time points were analyzed separately. No additional correction for multiple comparisons across these predefined time points was applied.

Data are presented as mean ± standard error of the mean (SEM). Statistical significance was defined as *p* < 0.05. Significance levels are indicated as follows: ns, *p* > 0.05; ^*^p ≤ 0.05; ^**^p ≤ 0.01; ^***^p ≤ 0.001; ^****^p ≤ 0.0001.

## Results

### Cortical neurons express the FcγRIIb and FcRn

Our first objective was to determine whether primary cortical neurons derived from rat brains expressed FcγRIIb and FcRn, the principal therapeutic targets of immunoglobulins and Efgartigimod, respectively. To establish a proinflammatory environment, neuronal cultures were exposed to conditioned medium from LPS-stimulated microglia, which secrete various proinflammatory mediators, including cytokines, chemokines, and reactive oxygen species (Hyvärinen et al., 2019). Immunocytochemical staining demonstrated FcγRIIb and FcRn immunoreactivity in MAP2-positive cortical neurons *in vitro* under both control and proinflammatory conditions (Figures 1a–d).

**Figure 1:**
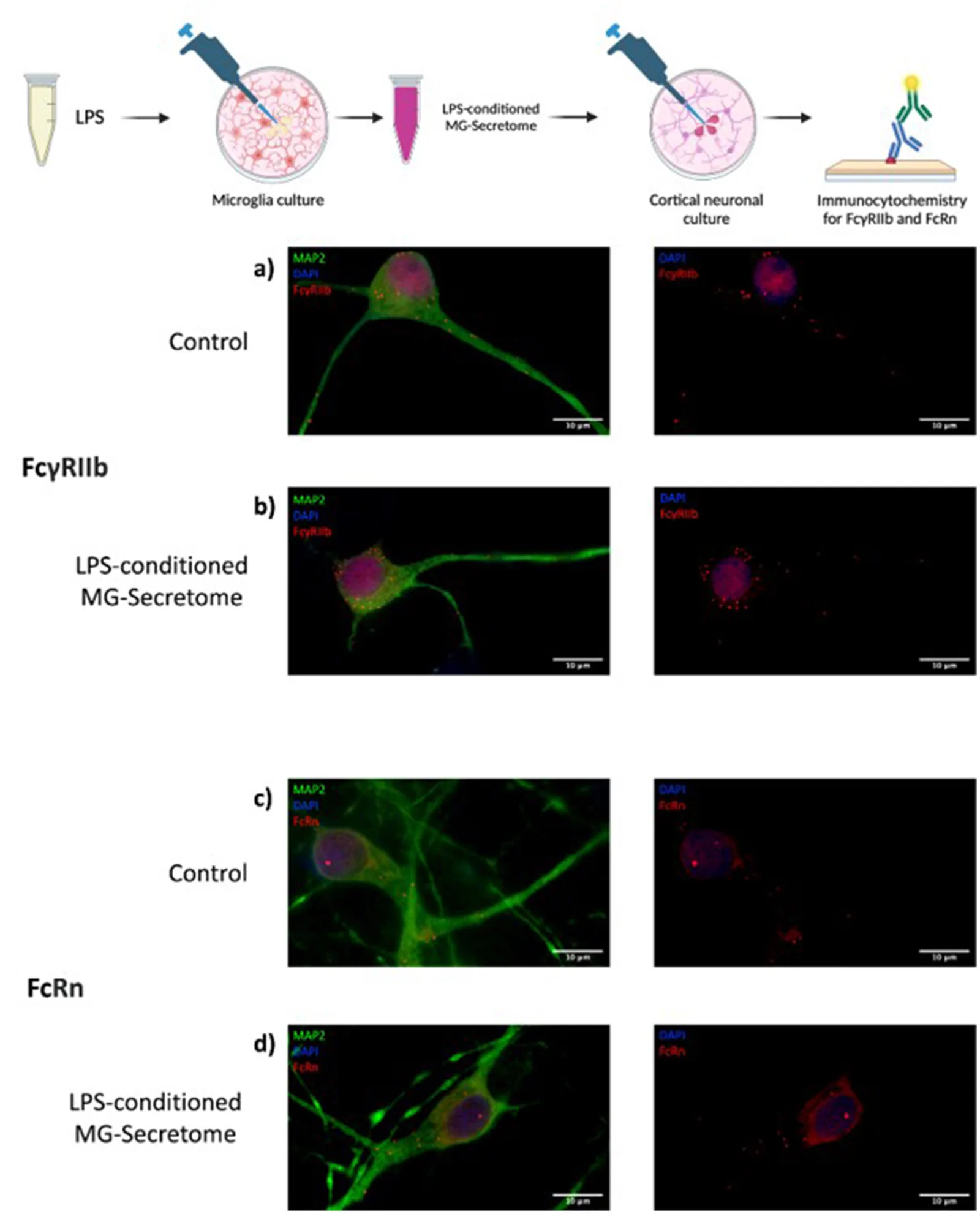
Cortical neurons show immunoreactivity for FcγRIIb and the neonatal Fc receptor FcRn. A schematic illustration of the induction of a proinflammatory milieu for neurons *in vitro* is shown in the upper part of the figure**. (a–d)** Representative immunocytochemical stainings of untreated neurons (control) and neurons cultured in a proinflammatory environment. Neurons were identified by MAP2 staining (green). Cell nuclei were counterstained with DAPI (blue). **(a, b)** Immunocytochemical staining of FcγRIIb (red); **(c, d)** staining of FcRn (red). Representative images are shown; the corresponding quantitative analysis is presented in Figure 2a. Scale bars = 10 μm.

### An inflammatory milieu increases FcγRIIb immunoreactivity in cortical neurons

To assess whether FcγRIIb and FcRn expression was affected by inflammatory conditions, we quantified their immunoreactivity in MAP2-positive neurons under homeostatic vs. proinflammatory conditions. Upon stimulation with media from microglia that had been exposed to LPS, FcγRIIb immunoreactivity increased significantly, with an average of 62.5 receptors per neuron compared to 35.6 in control cultures (1.76-fold increase). In contrast, FcRn immunoreactivity remained unchanged (35.2 vs. 34.8 receptors per neuron; Figure 2a). Consistent with the immunocytochemical findings, analysis at the transcript level showed a similar pattern, where Fcgr2b (FcγRIIb) expression was strongly upregulated (9.59-fold), in contrast to Fcgrt (FcRn), which showed no significant change (Figure 2b).

**Figure 2:**
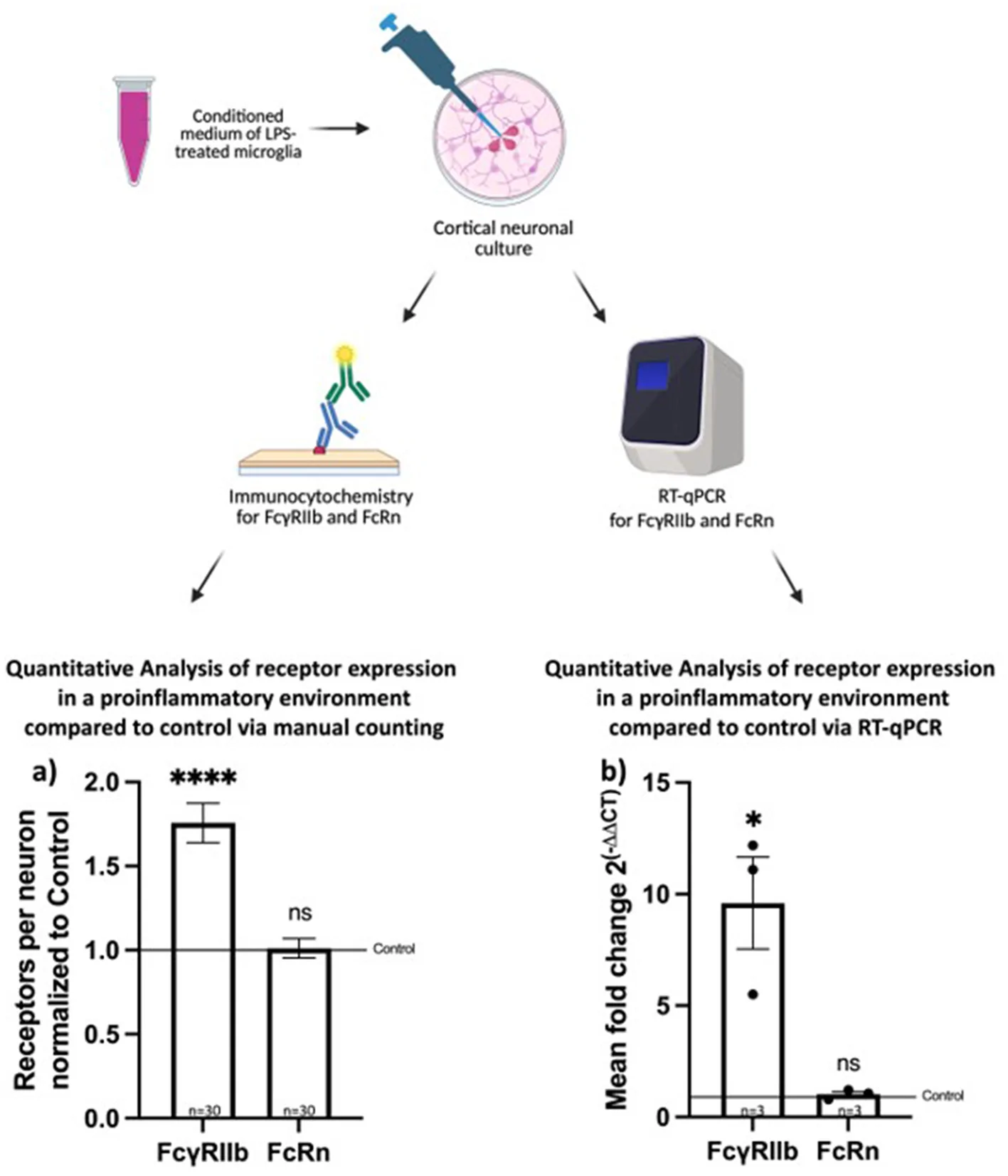
A proinflammatory milieu increases FcγRIIb immunoreactivity in cortical neurons and FcγRIIb transcript levels in cortical neuronal cultures. A schematic overview of the induction of a proinflammatory environment for neurons, along with the applied analytical methods, is shown in the upper part of the figure. **(a)** Quantification of FcγRIIb and FcRn receptor-specific immunoreactivity in MAP2-positive neurons under proinflammatory conditions compared to control, based on manual counting of receptor-specific immunocytochemical stainings (see representative images in Figure 1). Statistical comparisons were performed using two-tailed Mann–Whitney U tests. **(b)** Gene expression analysis of FcγRIIb and FcRn under proinflammatory conditions compared to control, assessed at the mRNA level. Statistical comparisons were performed using two-tailed unpaired t-tests. ns *p* > 0.05; *p ≤ 0.05; ****p ≤ 0.0001 compared to control group.

### Inflammation and immunoglobulins differentially shape cortical neuronal activity

Neurons were treated with conditioned medium from microglia that were either homeostatic or had undergone proinflammatory activation by LPS. At 4 h, neurons exposed to the proinflammatory milieu showed a significantly higher spike rate compared to those treated with homeostatic microglial medium (2.4-fold vs. 1.2-fold over baseline). By 24 hours, neuronal activity had largely converged between the two conditions, with no significant difference between neurons under control and proinflammatory conditions (1.5-fold vs. 1.7-fold, respectively; Figure 3a). Neither microglial culture medium itself nor conditioned medium from untreated microglia significantly affected neuronal activity compared with standard neuronal medium (Supplementary Figure 1).

**Figure 3:**
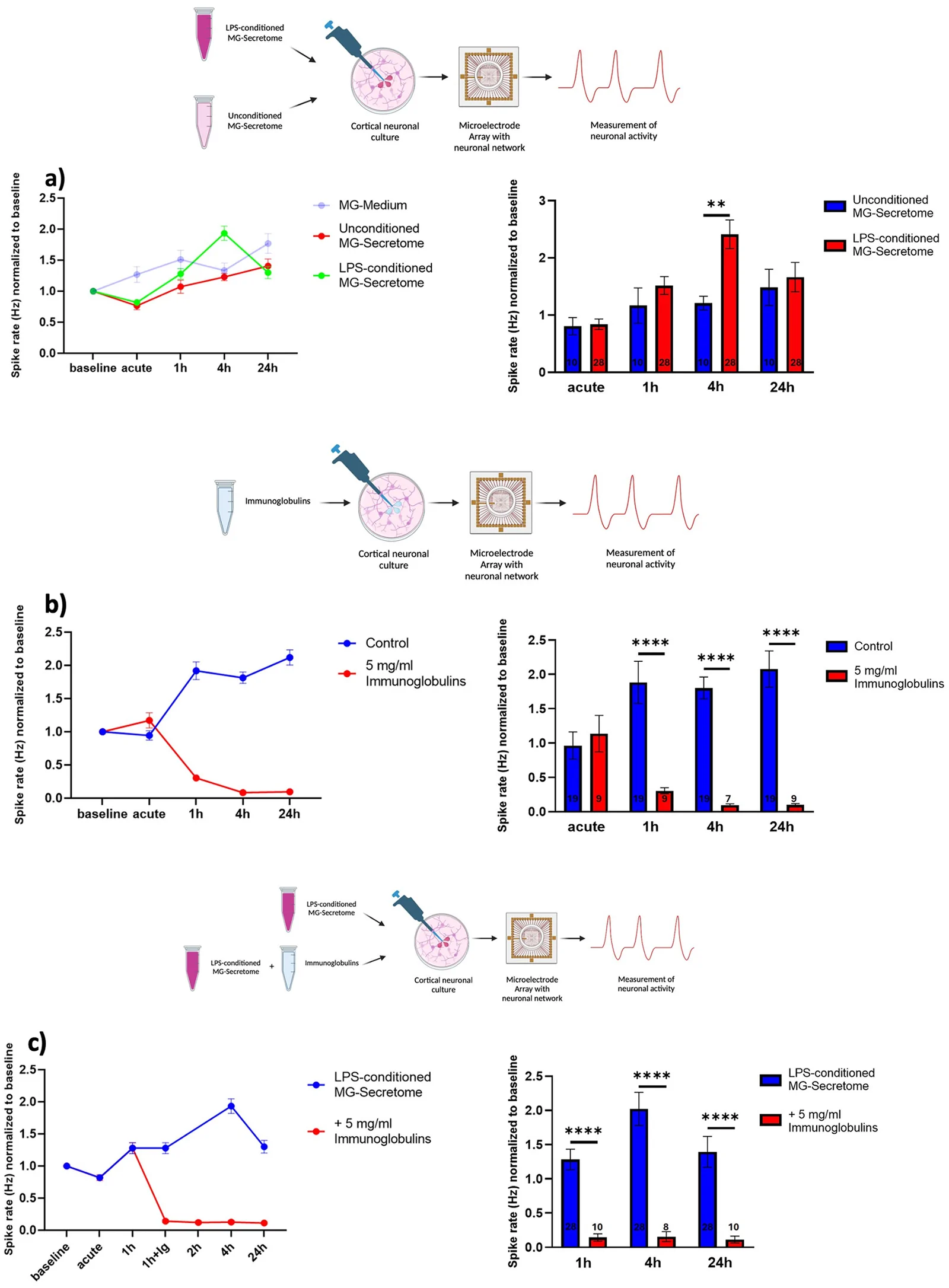
Inflammation and immunoglobulins differentially modulate cortical neuronal activity. A schematic overview of the respective treatment conditions applied before neuronal activity measurements is shown in the upper part of each panel **(a–c)**. Spike rate changes were recorded using microelectrode array (MEA) technology at different time points: **(a)** Neuronal activity in response to conditioned medium from untreated (homeostatic) or LPS-treated microglia. **(b)** Activity changes following stimulation with immunoglobulins (5 mg/ml). **(c)** Activity under inflammatory conditions (LPS-conditioned medium), with or without additional immunoglobulin treatment. Graphs depict mean spike rate normalized to baseline ± standard error of the mean (SEM). Left and right graphs in each panel represent the same data, displayed differently for improved visualization. Statistical comparisons between the indicated experimental groups at individual time points were performed using two-tailed Mann–Whitney U tests. No additional correction for multiple comparisons across the predefined time points was applied. ^*^p ≤ 0.05; ^**^p ≤ 0.01; ^****^p ≤ 0.0001, as indicated by horizontal bars. MEA experiments were performed using neuronal cultures derived from at least three independent neuronal preparations. Electrode-level measurements were averaged within each well, and statistical analyses were performed on well-level data. The number of wells (n) included in each experimental condition is indicated in the respective graphs.

We next examined how immunoglobulins affected cortical neuronal activity under physiological and inflammatory conditions. Note that in untreated cultures, neuronal activity showed a slight but steady increase over 24 h (Figure 3b, Figure 4a, blue line), reflecting synaptic plasticity and network maturation *in vitro* at this developmental stage (15–20 days). Immunoglobulin treatment strongly reduced neuronal activity under physiological conditions, with spike rates reduced to 0.3-fold at 1 h, 0.1-fold at 4 h, and at 24 h (Figures 3b). As an additional plasma protein comparator, neurons were also exposed to albumin. Albumin likewise reduced neuronal activity, although to a significantly lesser extent than immunoglobulins (supplementary Figure 2). Immunoglobulins also reduced neuronal activity in a proinflammatory environment when applied 1 h after stimulation with conditioned medium from LPS-activated microglia (Figure 3c).

**Figure 4:**
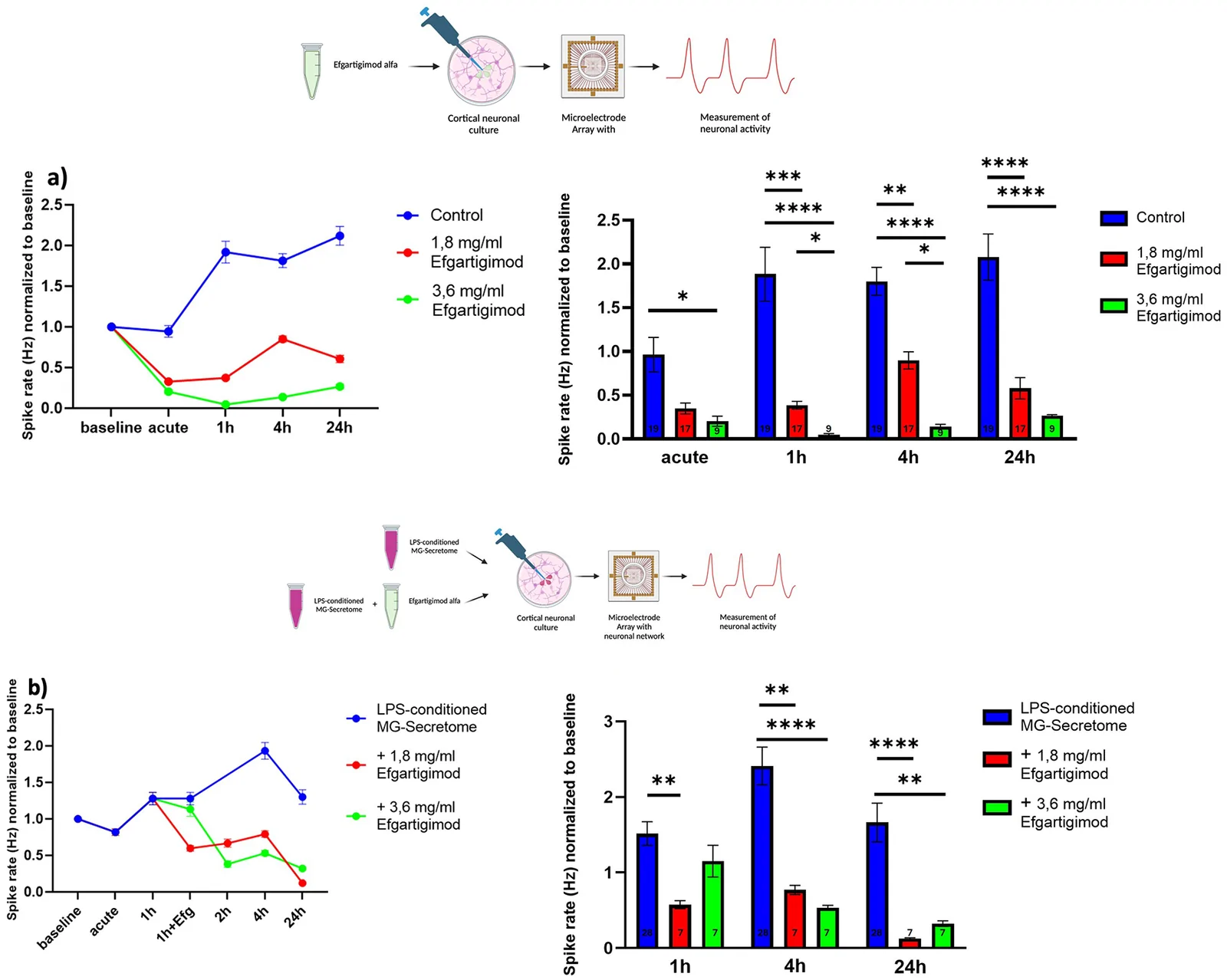
Efgartigimod alfa leads to a decrease in neuronal activity. A schematic overview of the respective treatment conditions applied before neuronal activity measurements is shown in the upper part of each panel **(a, b)**. Spike rate changes were recorded using microelectrode array (MEA) technology at different time points: **(a)** Neuronal activity in response to Efgartigimod alfa at two concentrations (1.8 and 3.6 mg/ml) under physiological conditions, compared to untreated controls. **(b)** Neuronal activity under inflammatory conditions (LPS-conditioned microglial medium), with or without additional treatment with Efgartigimod alfa. Graphs depict mean spike rate normalized to baseline ± standard error of the mean (SEM). Left and right graphs in each panel represent the same data, displayed differently for improved visualization. Statistical comparisons between the experimental groups at individual time points were performed using Kruskal–Wallis tests followed by Dunn's multiple-comparisons tests. Adjusted p values from Dunn's multiple-comparisons tests are shown. No additional correction across the predefined experimental time points was applied. ns *p* > 0.05; *p ≤ 0.05; **p ≤ 0.01; ***p ≤ 0.001; ****p ≤ 0.0001, as indicated by horizontal bars. MEA experiments were performed using neuronal cultures derived from at least three independent neuronal preparations. Electrode-level measurements were averaged within each well, and statistical analyses were performed on well-level data. The number of wells (n) included in each experimental condition is indicated in the respective graphs.

### Efgartigimod alfa reduces neuronal activity under physiological and inflammatory conditions

Next, we investigated a more targeted modulation of neuronal Fc receptors beyond the broad effects of immunoglobulins. To this end, we assessed the impact of Efgartigimod alfa, an IgG1-derived Fc fragment that binds specifically and with high affinity to FcRn (Ulrichts et al., 2018), on cortical neuronal activity. Two concentrations of Efgartigimod were tested: 1.8 mg/ml, approximating the Fc fragment content in 5 mg/ml of immunoglobulins, and 3.6 mg/ml to evaluate potential dose-dependent effects.

We first examined the effects of Efgartigimod alfa in a physiological environment, comparing treated neurons to control neurons that received a simple medium change. Neurons exposed to Efgartigimod showed an immediate decrease in spike activity: 0.33-fold relative to baseline at 1.8 mg/ml, and 0.20-fold at 3.6 mg/ml (Figure 4a). The reduction in neuronal activity persisted for at least 24 hours, showing only mild reversibility, with spike rates at 0.61-fold and 0.27-fold of baseline, respectively. In contrast, as previously shown, untreated controls displayed a physiological increase in activity over the same period (2.12-fold compared to baseline; Figure 4a). The greater reduction observed at the higher Efgartigimod concentration indicated a trend toward a concentration-dependent effect under control conditions.

Finally, similar to the results observed with immunoglobulins, Efgartigimod alfa maintained its rapid and sustained inhibitory effect on neuronal activity even in a proinflammatory environment (Figure 4a). In contrast to the concentration-dependent trend observed under control conditions, no consistent concentration-dependent pattern was observed under proinflammatory conditions.

## Discussion

Our study provides evidence for FcγRIIb expression in primary rat cortical neurons. This is in line with the few previous studies that reported its expression in neurons of mice (Fernandez-Vizarra et al., 2012), humans (Kam et al., 2013), and rats (Stamou et al., 2018), both in the cortex and the hippocampus. Building upon these findings, we show that FcγRIIb immunoreactivity in MAP2-positive cortical neurons is increased in response to a proinflammatory environment—a phenomenon that, to the best of our knowledge, has not previously been reported. Although FcγRIIb upregulation in neurons has been observed in other contexts—for example, following amyloid-β exposure (Kam et al., 2013)—a direct link between inflammatory stimuli and increased FcγRIIb immunoreactivity in cortical neurons has not previously been described. Notably, inflammation-associated upregulation of FcγRIIb has also been reported in glial cells (Han et al., 2024). Since the neuronal cultures used in the present study were not completely depleted of non-neuronal cells, a potential contribution of residual glial cells, particularly to the transcript-level measurements, cannot be excluded. Nevertheless, the increased FcγRIIb immunoreactivity observed specifically in MAP2-positive cells supports inflammation-associated regulation of FcγRIIb in cortical neurons. The mechanisms driving this upregulation remain to be elucidated. Nevertheless, our results provide new insights into the complex interplay between neuronal and immune signaling and suggest that neuronal FcγRIIb may contribute to neuronal responses under inflammatory conditions.

We also found evidence for the expression of the neonatal Fc receptor (FcRn) in neurons, a finding that has remained controversial. Latvala et al. observed only low levels in a humanized mouse model (Latvala et al., 2017), and Stamou et al. were unable to detect FcRn in primary rat neurons (Stamou et al., 2018). Similarly, Niu et al. identified FcRn exclusively in microvascular endothelial cells of the human CNS (Niu et al., 2011), where it mediates IgG transport across the blood–brain barrier (Schlachetzki et al., 2002; Zhang and Pardridge, 2001). Moreover, data from publicly available single-cell transcriptomic databases support our detection of FcRn in cortical neurons. Analysis of the BrainCell Atlas (Saunders et al., 2018) indicates that Fcgrt is expressed—albeit at low levels—in several neuronal populations within the cerebral cortex of mice. In agreement with these observations, we detected FcRn immunoreactivity in MAP2-positive cortical neurons, while Fcgrt transcripts were additionally detected in the cortical neuronal cultures. The detection of FcRn in cortical neurons provides a strong rationale for further investigations into its potential role in neuroimmune regulation and IgG dynamics within the brain. However, given the possible presence of residual non-neuronal cells in our cultures, cell-type-specific approaches will be required to further establish the distribution and functional relevance of FcRn in cortical neurons.

A key finding of this study is that an inflammatory milieu transiently alters neuronal activity. To model this condition, neurons were treated with the conditioned medium of LPS-activated microglia. LPS stimulation induces a well-characterized proinflammatory phenotype in microglia, leading to the release of cytokines such as IL-1α, IL-1β, IL-6, IL-12, IL-23, TNF-α, along with chemokines, redox molecules, scavenger receptors, costimulatory molecules, and MHC-II (Orihuela et al., 2016). The effects of these proinflammatory mediators on neuronal activity remain controversial. While Wegrzyn et al. reported an excitatory effect on hippocampal neurons (Wegrzyn et al., 2021), Vakili et al. observed reduced action potential firing in rat hippocampal neurons following exposure to extracellular vesicles from M1 macrophages (Vakili et al., 2020). Similarly, the impact of TNF-α on neuronal activity is conflicting: Hagman et al. described inhibitory effects on human neurons (Hagman et al., 2019), whereas Gullo et al. found increased activity in murine cortical neurons (Gullo et al., 2014). IL-6 has also yielded inconsistent results — Mzezewa et al. observed no effect on human cortical neurons (Mzezewa et al., 2022), while Black et al. reported enhanced activity in sensory dorsal root ganglion neurons in mice (Black et al., 2018). Given these inconsistencies, there is no clear consensus regarding the net effect of inflammatory cytokines on neuronal excitability. However, our findings indicate that an inflammatory environment can transiently enhance neuronal activity in cortical cultures. Future studies are warranted to explore the effects of individual cytokines or defined cytokine mixtures to dissect their specific contributions to neuronal network modulation.

These functional findings raise important mechanistic questions. We demonstrated that both immunoglobulins and Efgartigimod alfa reduced neuronal activity. This is the first study to directly measure and compare the effects of both agents on neuronal activity. Previous experiments performed under comparable immunoglobulin treatment conditions did not indicate increased cell death, supporting the interpretation that the reduction in neuronal activity following immunoglobulin exposure is not primarily attributable to overt cytotoxicity (Supplementary Figure 3). Interestingly, albumin, another physiological ligand of FcRn, also reduced neuronal activity, although to a lesser extent than immunoglobulins. However, albumin should be considered an additional plasma protein comparator rather than a formulation- or osmolarity-matched control. Thus, nonspecific effects related to the applied protein formulations, including potential contributions from formulation excipients, viscosity, or other physicochemical properties, cannot be fully excluded. Formulation-matched vehicle controls would therefore be valuable in future mechanistic studies. Nevertheless, the expression of FcγRIIb and FcRn in cortical neurons raises the possibility that Fc receptor-associated mechanisms contribute to the observed effects. Stamou et al. showed that IgG immune complexes increased intracellular calcium levels in primary rat cortical and hippocampal neurons. They proposed that this effect was likely due to a direct interaction of immunoglobulins with Fcγ receptors, such as FcγRIIb, on neurons. However, FcRn expression was not detected in their neuronal cultures (Stamou et al., 2018). An increase in intracellular calcium influences neuronal activity by activating calcium-dependent potassium channels that play a key role in the repolarization phase of action potentials, and may lead to membrane hyperpolarization (Bean, 2007; Nguyen et al., 2007). This would inhibit action potential firing and thereby reduce neuronal activity and could therefore represent one possible mechanism contributing to the reduced neuronal activity observed in the present study. The observation that Efgartigimod alfa, which contains an IgG1-derived Fc fragment and binds FcRn with high affinity (Ulrichts et al., 2018), also reduced neuronal activity raises the possibility that FcRn-associated processes may contribute to the observed response. However, the specific role of FcRn — for which Efgartigimod has an increased binding affinity—remains unresolved. In non-neuronal cells, FcRn is known to mediate IgG recycling via transcytosis (Roopenian and Akilesh, 2007). It is conceivable that the internalization of immunoglobulins or Fc-based biologics via FcRn could also influence intracellular signaling in neurons. Stamou et al. demonstrated that immunoglobulins can be internalized into neurons via Fcγ receptor–mediated mechanisms, an effect attenuated when FcγRIa and FcγRIIb are inhibited (Stamou et al., 2018). These findings suggest that Fc receptor–mediated internalization represents one possible mechanism underlying neuronal responses to immunoglobulins, although its functional relevance remains to be determined. Interestingly, the trend toward a concentration-dependent effect of Efgartigimod observed under control conditions was not apparent under proinflammatory conditions. Given the exploratory nature of the study, the underlying mechanisms remain unclear and warrant further investigation. In addition, as the inflammatory challenge preceded immunoglobulin exposure in our experimental paradigm, future studies may investigate whether the order of stimulation influences the neuronal response.

Another limitation concerns the relatively high concentrations of immunoglobulins and Efgartigimod used *in vitro*, which are unlikely to reflect neuronal exposure *in vivo*. However, the primary aim of this proof-of-concept study was not to model clinically relevant CNS concentrations, but to investigate whether neuronal Fc receptors can exert functional effects on neuronal activity. Future studies using more physiologically relevant concentrations will be required to determine the potential *in vivo* relevance of these findings.

Altogether, our findings provide proof-of-concept evidence that immunoglobulins and Efgartigimod can modulate neuronal network activity *in vitro* and suggest that neuronal Fc receptor-associated mechanisms represent an interesting direction for further mechanistic investigation.

## Conclusion

Our findings represent an *in vitro* proof-of-concept providing evidence for FcγRIIb and FcRn expression in cortical neurons and demonstrating changes in neuronal activity following exposure to immunoglobulins and Efgartigimod. This work highlights a previously underexplored interface between immunoglobulin signaling and neuronal activity. The observed effects of immunoglobulins and Efgartigimod under both physiological and inflammatory conditions provide a rationale for further investigating the potential contribution of neuronal Fc receptor-associated mechanisms to neuroimmune communication. Further *in vivo* studies and disease models are required to determine the underlying mechanisms and assess their physiological and clinical relevance.

## Data Availability

The original contributions presented in the study are included in the article/Supplementary material, further inquiries can be directed to the corresponding author.

## Supplementary material

Supplementary Figure 1 Neuronal activity under control conditions. Spike rate changes were recorded using microelectrode array (MEA) technology at different time points. Left: neuronal activity in response to unconditioned microglial culture medium compared with control neurons maintained in Neurobasal medium. Right: neuronal activity in response to conditioned medium from untreated microglia compared with unconditioned microglial culture medium.

Supplementary Figure 2 Albumin reduces neuronal activity to a lesser extent than immunoglobulins. Spike rate changes were recorded using microelectrode array (MEA) technology at different time points: Activity changes following stimulation with immunoglobulins (5 mg/ml) or albumin (5 mg/ml).

Supplementary Figure 3 Neuronal viability following treatment with different concentrations of immunoglobulins. Neurons were treated for 24 h with immunoglobulins at concentrations of 0, 100, 250, and 500 5g/ml and 1 and 5 mg/ml. All cells were stained with Hoechst (blue), and dead cells with propidium iodide (red). **(a)** Untreated neurons. **(b)** 100 5g/ml. **(c)** 250 5g/ml. **(d)** 500 5g/ml. **(e)** 1 mg/ml. **(f)** 5 mg/ml. Scale bars: 50 5m. **(g)** Quantification of neuronal survival following treatment with the indicated immunoglobulin concentrations. Data are presented as mean ± SEM.

Supplementary Figure 4 Representative MEA traces illustrate changes in spontaneous neuronal activity following exposure to proinflammatory conditioned medium, immunoglobulins, and Efgartigimod alfa. Representative microelectrode array (MEA) traces of spontaneous neuronal activity are shown at baseline and 4 h after the respective treatment. Each trace represents the recording from a single representative electrode of a 60-electrode MEA from one independent neuronal culture. **(a)** Neuronal activity at baseline and 4 h after exposure to conditioned medium from LPS-treated microglia. **(b)** Neuronal activity at baseline and 4 h after treatment with immunoglobulins (5 mg/ml). **(c)** Neuronal activity at baseline and 4 h after treatment with Efgartigimod alfa (3.6 mg/ml). Quantitative analyses based on electrode-level measurements from at least three independent neuronal cultures per experimental condition are presented in Figure 3 and Figure 4.
