# Supplementary material for: Immunoglobulins and Efgartigimod reduce neuronal activity in primary rat cortical neuron cultures
Source: Front Cell Neurosci. 2026 Oct 28;20:1939772. doi: 10.3389/fncel.2026.1939772 (PMC13751231; doi:10.3389/fncel.2026.1939772)
Supplement: Supplementary file 1 [file Image_4.pdf]

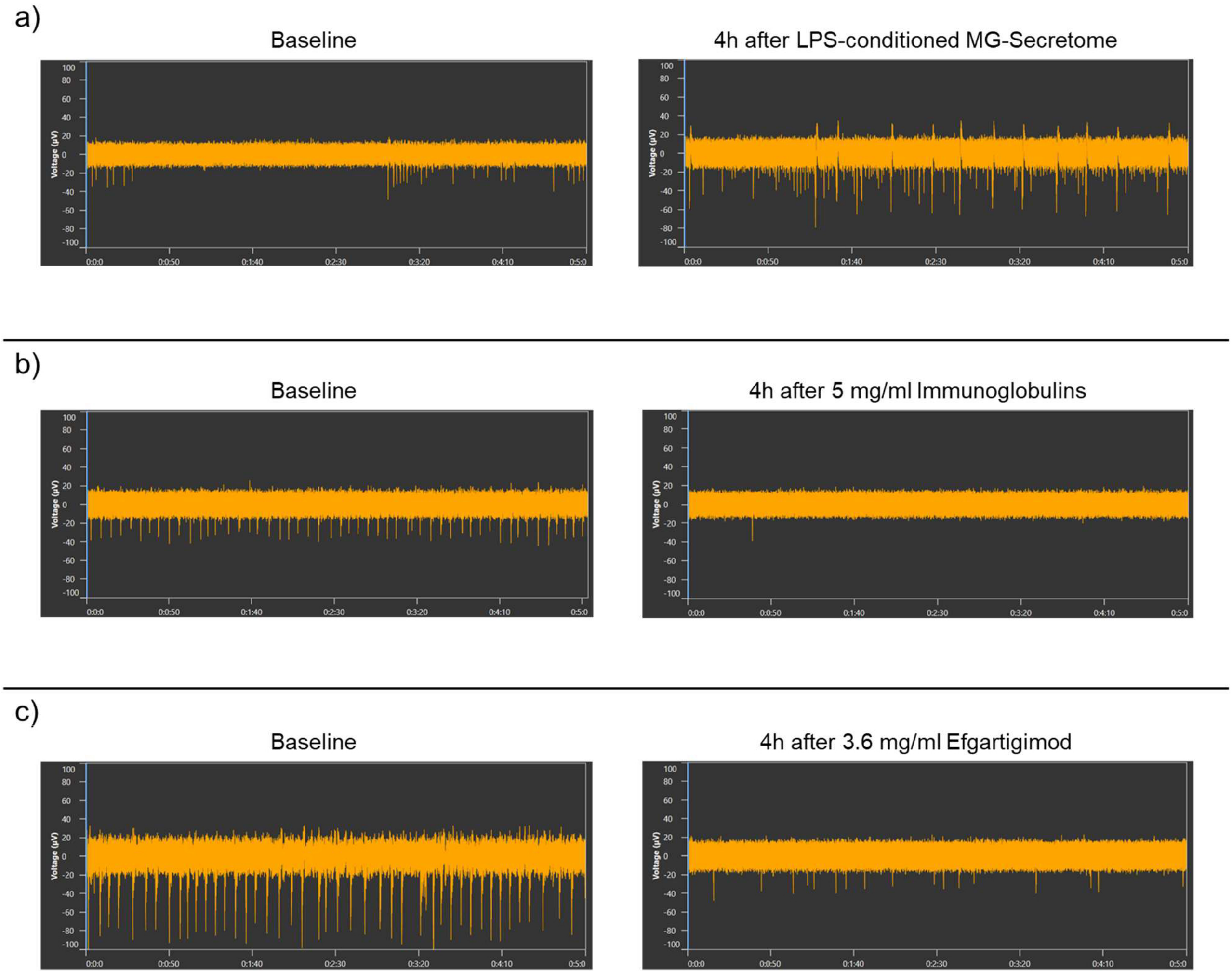
a)
Baseline
4h after LPS-conditioned MG-Secretome
b)
Baseline
4h after 5 mg/ml Immunoglobulins
c)
Baseline
4h after 3.6 mg/ml Efgartigimod
Voltage (µV)
100 80 60 40 20 0 -20 -40 -60 -80 -100
0:0:0 0:0:50 0:1:40 0:2:30 0:3:20 0:4:10 0:5:0
